# Capturing the True Cost of Breast Cancer Treatment: Molecular Subtype and Stage-Specific per-Case Activity-Based Costing

**DOI:** 10.3390/curroncol30090571

**Published:** 2023-08-26

**Authors:** Anna N. Wilkinson, Jean M. Seely, Moira Rushton, Phillip Williams, Erin Cordeiro, Alexandra Allard-Coutu, Nicole J. Look Hong, Nikitha Moideen, Jessica Robinson, Julie Renaud, James G. Mainprize, Martin J. Yaffe

**Affiliations:** 1 Department of Family Medicine, Faculty of Medicine, University of Ottawa, Ottawa, ON K1H 8L6, Canada; 2 Department of Radiology, The Ottawa Hospital Research Institute, University of Ottawa, Ottawa, ON K1H 8L6, Canada; jeseely@toh.ca; 3 The Ottawa Hospital Cancer Centre, 501 Smyth Rd., Ottawa, ON K1H 8L6, Canada; moirushton@toh.ca (M.R.); nmoideen@toh.ca (N.M.); jessrobinson@toh.ca (J.R.); jurenaud@toh.ca (J.R.); 4 Division of Anatomic Pathology, The Ottawa Hospital, 501 Smyth Rd., Ottawa, ON K1H 8L6, Canada; philwilliams@eorla.ca; 5 Division of General Surgery, Department of Surgery, University of Ottawa, Ottawa, ON K1H 8L6, Canada; ecordeiro@toh.ca (E.C.); aallardcoutu@toh.ca (A.A.-C.); 6 Department of Surgery, University of Toronto, Toronto, ON M5T 1P5, Canada; nicole.lookhong@sunnybrook.ca; 7 Department of Medical Biophysics, University of Toronto, Toronto, ON M4N 3M5, Canada; james.mainprize@sri.utoronto.ca (J.G.M.); martin.yaffe@sri.utoronto.ca (M.J.Y.)

**Keywords:** breast cancer, treatment, health economics, activity-based costing

## Abstract

Background: Breast cancer (BC) treatment is rapidly evolving with new and costly therapeutics. Existing costing models have a limited ability to capture current treatment costs. We used an Activity-Based Costing (ABC) method to determine a per-case cost for BC treatment by stage and molecular subtype. Methods: ABC was used to proportionally integrate multidisciplinary evidence-based patient and provider treatment options for BC, yielding a per-case cost for the total duration of treatment by stage and molecular subtype. Diagnostic imaging, pathology, surgery, radiation therapy, systemic therapy, inpatient, emergency, home care and palliative care costs were included. Results: BC treatment costs were higher than noted in previous studies and varied widely by molecular subtype. Cost increased exponentially with the stage of disease. The per-case cost for treatment (2023C$) for DCIS was C$ 14,505, and the mean costs for all subtypes were C$ 39,263, C$ 76,446, C$ 97,668 and C$ 370,398 for stage I, II, III and IV BC, respectively. Stage IV costs were as high as C$ 516,415 per case. When weighted by the proportion of molecular subtype in the population, case costs were C$ 31,749, C$ 66,758, C$ 111,368 and C$ 289,598 for stage I, II, III and IV BC, respectively. The magnitude of cost differential was up to 10.9 times for stage IV compared to stage I, 4.4 times for stage III compared to stage I and 35.6 times for stage IV compared to DCIS. Conclusion: The cost of BC treatment is rapidly escalating with novel therapies and increasing survival, resulting in an exponential increase in treatment costs for later-stage disease. We provide real-time, case-based costing for BC treatment which will allow for the assessment of health system economic impacts and an accurate understanding of the cost-effectiveness of screening.

## 1. Introduction

Approximately one in every eight women will be diagnosed with breast cancer (BC) over their lifetime, with an estimated 28,600 new cases of BC in Canada in 2022 [1,2]. BC treatment is rapidly evolving, with ongoing advances in genomic testing, radiation therapy, surgery and systemic therapy. BC molecular subtype, based on hormone receptor (HR) and HER2/neu (HER2) status, is increasingly driving specialised therapy choices and informing improved subtype-specific survival [3]. An accurate understanding of stage- and subtype-specific BC treatment cost is critical to accurately assess healthcare funding impacts and the cost-effectiveness of screening.

Approaches to BC treatment costing follow a top-down or gross costing method, or a bottom-up or micro-costing method, also known as Activity-Based Costing (ABC) [4]. Gross costing uses publicly available population-level data with aggregated costs, providing a system-level perspective, but may not capture indirect costs, privately paid drugs and treatment preferences when there are multiple therapeutic pathways [5]. Importantly, lags in data availability create delays in gross costing, such as in BC, where a 2021 study reported costs only from 2016 [6]. In the setting of BC, where treatments are rapidly evolving, any delay in informing costs limits the current applicability of such models.

ABC maps and identifies patient activities along a treatment trajectory, measuring resource use for each contact the patient has with the healthcare system [7,8]. Micro-costing allows for the inclusion of a broad range of sources of evidence and data to reflect current knowledge and practices around treatment algorithms and disease activity, and reflects clinician and patient preferences, giving a dynamic real-world perspective on an individual case basis. Micro-costing is particularly effective in settings with rapidly evolving treatments and tends to represent the true costs of medical care more accurately [9]. ABC outputs can be readily integrated into microsimulations such as OncoSim, which attempt to model the economic impacts of natural history, detection, diagnosis and treatment aspects of cancer management [10].

In this study, we use ABC to determine a per-case cost for BC by stage and molecular subtype, in Ontario, Canada. We proportionally integrate the range of multidisciplinary evidence-based patient and provider treatment options, to yield a single case cost for the total duration of treatment per case. The real-time, case-based nature of our model will allow for a better understanding of the actual cost of BC treatment and better modelling of health system economic impacts.

## 2. Methods

Treatment costs by stage at diagnosis and molecular subtype were determined by an expert panel with representation from radiology, pathology, surgery, radiation oncology, medical oncology, family medicine, pharmacy, cancer centre administration and cost modelling. The costing model can be found in Supplementary Table S1. The American Joint Committee on Cancer (AJCC) 8th edition staging system was used [11]. Molecular subtype was defined by HR and HER2 status: HR+/HER2−(HR+); HR+/HER2+; HR−/HER2+ (HER2+); HR−/HER2− (triple negative (TN)) [12]. Full case treatment costs, expressed in 2023 Canadian dollars, were modelled, from post-biopsy to completion of treatment (stages I–III) or until death (stage IV). The duration of treatment costs varied from less than one year for DCIS or stage I BC, to up to seven years of adjuvant endocrine therapy in stage III HR+. The duration of stage IV treatments was determined from summed published mean progression-free survival estimates and ranged from 22 to 66 months based on subtype (see systemic therapy methods). Costs were included for diagnostic imaging subsequent to biopsy, pathology, surgery, radiation therapy, systemic therapy, emergency room, home care, inpatient hospitalisation and palliative care where appropriate. Only publicly funded treatments in Ontario were considered. In the case of multiple treatment options, e.g., lumpectomy vs. mastectomy, treatments were proportionally weighted based on published data for frequency of use to generate a single cost. If there was a range of costs, e.g., a variable number of pathology blocks, the average cost was used. Pre- and post-menopausal status at diagnosis, proportions of molecular subtypes and proportions of T1b and T1c cases were derived from the Canadian Cancer Registry [13]. 

### 2.1. Costing Sources

Multiple cost sources were utilised and are detailed below by treatment category. For all cost categories, clinician professional fees, laboratory services and diagnostic imaging costs were obtained through the Ontario Health Insurance Plan (OHIP) schedule of benefits [14]. Drug costs were determined through the Ontario Drug Benefit Formulary [15], the New Drug Funding Program [16], Quality-Based Procedure (QBP) programs [17] or in the case of non-formulary medications, through the Canadian Agency for Drugs and Technologies in Health (CADTH) [18]. QBPs were included to allow for broader inclusion of all treatment costs. QBPs are funding bundles for defined healthcare services and include payment for nursing, clerical, dietician, social work, psychologist, pharmacist and administrator time as well as physical plant and supportive medications and equipment. Because QBPs and physician billing codes account for human resource use, it was not necessary to track these costs on a per-minute basis as in time-directed ABC models.

### 2.2. Diagnostic Imaging

The costs of screening or initial biopsy were not included. Imaging costs included the workup of BC beyond biopsy, including breast MRI and repeat biopsy when required. Assumptions about the proportions of patients undergoing these were determined by The Ottawa Hospital (TOH) practices (Supplemental Table S1). As per established guidelines in Ontario, staging investigations were not included for stages I or II BC, but a positron emission computed tomography scan (PET CT) was included for stage III [19]. Additional imaging for the investigation of clinical symptoms was not included in the costs for stage III. For stage IV cases, radiology costs included restaging investigations, consisting of a CT chest, abdomen and pelvis, assumed to occur every three months while on active treatment [20]. Bone scans, radiographs and brain imaging with CT and MRI were also included in radiological costs for stage IV cases.

### 2.3. Pathology

The number of pathology blocks was averaged from the range of blocks (17–39) used at TOH for invasive lesions at each stage. The cost per block included all the reagent costs, lab staff costs and pathologist time. Pathology costs also incorporated immunohistochemistry testing for HR and HER2 testing, with additional confirmational testing in HR- and HER2- cases. Fluorescence in situ hybridisation HER2 testing was assumed to be performed in 20% of cases with equivocal immunohistochemistry [21]. Ki67 was not routinely performed. Genomic testing with Oncotype Dx was assumed in half of stage I and stage II post-menopausal patients [22,23]. 

### 2.4. Surgery

**Figure 1:**
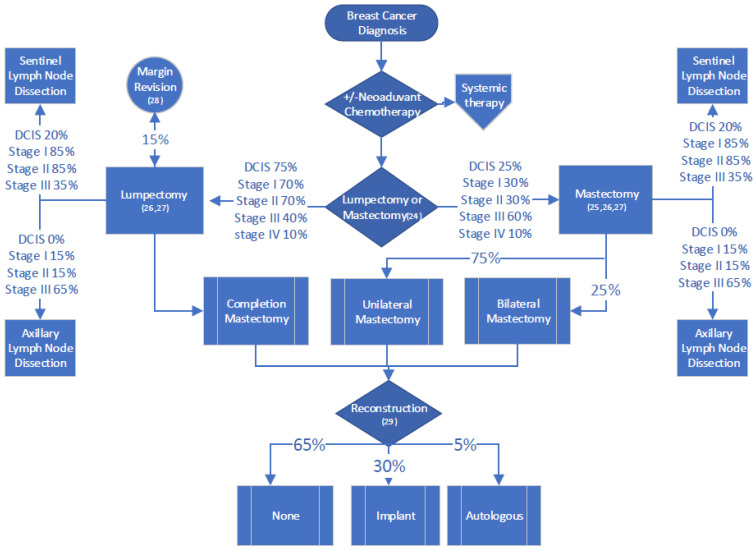
Surgical activity map. Percentages are proportions of patients who receive each specific treatment at each stage.

Surgical management of BC was largely independent of HR and HER2 status, and was contingent on the assessment of tumour size, nodal involvement with cancer and patient preference. Surgical options included lumpectomy or mastectomy [24], either unilateral or bilateral [25], along with sentinel node biopsy or axillary lymph node dissection [26,27]. Post-lumpectomy, it was assumed that there may be a need for additional surgery for margin revision, or for a completion mastectomy, depending on pathology results [28]. It was assumed that a proportion of patients post-mastectomy would decide to have breast reconstructive surgery with either implant or autologous reconstruction [29]. Surgical costing proportionally integrated the costs of the varying types of surgeries possible for each stage of disease (Figure 1). Contemporary rates of surgical procedures were derived from the Canadian context, recognizing that the rates of lumpectomies, unilateral and bilateral mastectomies may vary between countries. QBP funding for BC surgery encompassed presurgical assessment, the pre-operative care unit, operating room and post-anaesthetic care unit costs, as well as costs incurred on the first post-operative day [17]. Clinician billings were included for the surgical procedure, anaesthetic administration and a post-operative follow-up visit [14]. Delayed breast reconstruction and prophylactic surgeries due to documented gene mutations were not incorporated into this model. 

### 2.5. Radiation Therapy

**Figure 2:**
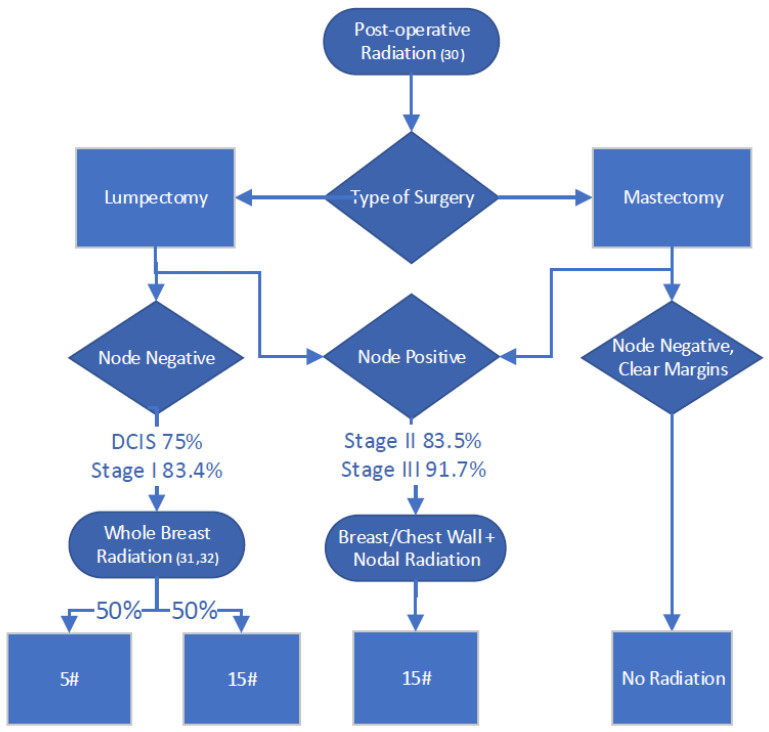
Radiation therapy activity map. Percentages are proportions of patients who receive each specific treatment at each stage.

Adjuvant radiation therapy (RT) was assumed to be offered following lumpectomy and for select post-mastectomy patients (≥T3, and/or positive nodes/margins) [30]. Fractionation regimens reflected the shift in clinical practice from conventionally fractionated (50 Gray in 25 fractions) to hypofractionated (40–42.5 Gray in 15–16 fractions) and, more recently, ultra-hypofractionated treatment (26 Gray in 5 fractions) [31,32]. The proportion of stage I–III patients treated with each fractionation scheme was derived from practice patterns at TOH. In metastatic disease, the proportion of site- and subtype-specific metastases directed RT was derived from the published literature (Figure 2) [33]. Costs for simulation, dosimetry, treatments and follow-up visits were included within the QBP bundle [17]. Clinician billings were included for consult and follow-up visits [14]. The capital equipment and ownership costs of linear accelerators were not included, but machine maintenance and upkeep were factored into the assumptions.

### 2.6. Systemic Therapy

Costing assumptions for systemic treatment regimes were based on published guidelines from Cancer Care Ontario (CCO) [34], the American Society of Clinical Oncology (ASCO) [35] and funding guidelines from CADTH [36]. Time on treatment was based on mean progression-free survival (PFS) published in phase 3 clinical trials as detailed below. Clinician billings for consults, follow-up and monthly supervision of oral, endocrine or chemotherapy, as well as palliative care oversight, were included [14]. QBP funding includes nursing, pharmacist and allied health costs, as well as some supportive medications [17]. Lab costs for pre-treatment bloodwork monitoring were included, as were echocardiograms for patients on anthracyclines and trastuzumab [14].

#### 2.6.1. HR+ 

All patients with early-stage HR+ BC were assumed to be treated with endocrine therapy. Node-negative patients were assumed to receive 5 years of therapy: pre-menopausal patients with tamoxifen and post-menopausal patients with a tamoxifen/aromatase inhibitor (AI) switch strategy [37]. Node-positive patients were assumed to receive 7 years of endocrine therapy, although there may be a subset of very high-risk patients that receive up to 10 years of hormonal therapy [38].

Adjuvant chemotherapy was assumed for high-risk stage I and II patients (based on clinical and genomic assessments) and all stage III patients [22,36,39]. Stage I high-risk patients were assumed to be treated with four cycles of cyclophosphamide/docetaxel, while stage II/III patients were assumed to be treated with eight cycles of dose-dense doxorubicin/cyclophosphamide followed by paclitaxel [40].

Ovarian suppression was assumed for pre-menopausal stage III patients [41]. Two years of the adjuvant CDK4/6 inhibitor, abemaciclib, were included for patients with stage III disease [42]. All post-menopausal patients who received chemotherapy were also assumed to receive adjuvant bisphosphonates [43].

For stage IV HR+ BC, first-line (1L) therapy was with a CDK4/6 inhibitor (e.g., ribociclib) and AI [44]. Second-line (2L) treatment was assumed to be Fulvestrant [45]. There is currently no publicly funded access to PIK3CA inhibitors or oral SERDs in Canada [36]. Third-line (3L) and beyond therapy was with single-agent chemotherapy, while treatment with trastuzumab deruxtecan in the fourth line (4L) was assumed for HER2-low patients [46]. 

#### 2.6.2. HER2+ and HR+/HER2+

Patients with T1b and T1c disease were assumed to be treated with weekly paclitaxel + trastuzumab [47]. All patients with stage II and III HER2+ BC were assumed to receive neoadjuvant chemotherapy with HER2-targeted therapy [48], and the estimated 50% of patients with residual disease were treated with 14 cycles of adjuvant trastuzumab emtansine [36,39]. Pertuzumab was not included as it is not funded in the curative setting in Ontario. Standard adjuvant hormonal therapy for HR+ and bisphosphonates were also included in cost estimates.

Metastatic HER2+ BC was treated with sequential lines of treatment, including 1L taxane/trastuzumab/pertuzumab, 2L trastuzumab deruxtecan and 3L capecitabine/tucatinib/trastuzumab [36,49]. Treatment beyond 3L is only funded as single-agent chemotherapy. For patients with triple-positive disease, concurrent treatment with tamoxifen or AI was assumed for 50% of overall survival. 

#### 2.6.3. Triple Negative

Patients with TNBC T1b or greater were assumed to be treated with chemotherapy, while patients with stages II and III also received pembrolizumab [50]. For the approximately 40% that do not have a pathological complete response to this regimen, adjuvant capecitabine was also included in treatment assumptions [48]. Adjuvant olaparib is not publicly funded in Canada.

In metastatic TNBC, 1L pembrolizumab with chemotherapy was included for 30% of patients with a combined positive score (number of PDL-L1 staining tumour cells, lymphocytes and macrophages divided by the total number of viable tumor cells, multiplied by 100) >10 [51] and 2L sacituzumab govitecan was assumed for all patients [52]. Trastuzumab deruxtecan was assumed as 3L for HER2-low patients [46]; subsequent treatments were assumed to be single-agent chemotherapy. 

### 2.7. Inpatient, Emergency Room and Home Care Costs

The cost of inpatient hospitalisations, emergency department visits and home care use from Ontario Ministry of Health and Long-Term Care databases (the National Ambulatory Care Reporting System, The Ontario Association of Community Care Access Centers and the Discharge Abstract Database) were obtained from a previous study [53]. These costs, representing a two-year period, were updated to 2023 dollars using the consumer price index [54].

### 2.8. Derivation of Per-Case Cost

Costs for post-biopsy radiological investigations, pathology, surgery, radiation therapy, systemic therapy, emergency room, home care, inpatient hospitalisation and palliative care were summed for each molecular subtype and stage to arrive at a per-case cost.

## 3. Results

**Figure 3:**
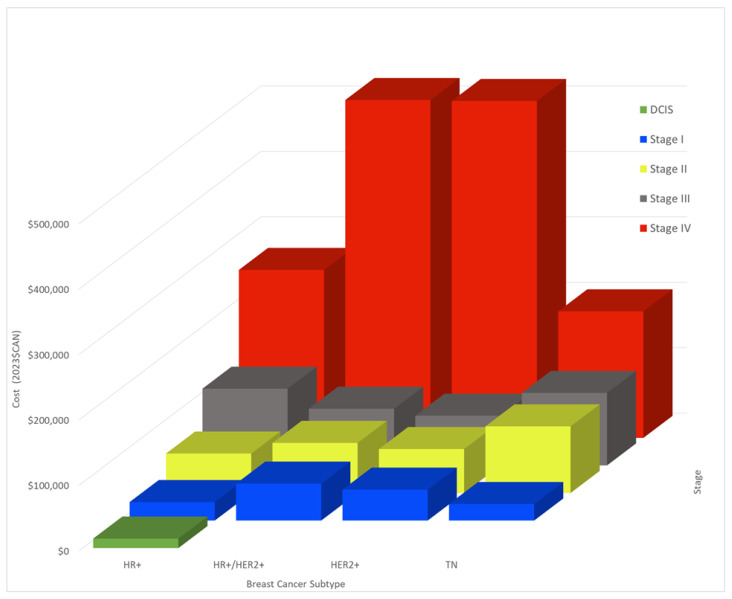
Breast cancer per-case cost in C$ of treatment by stage and molecular subtype.

The costing model can be found in Supplementary Table S1. The per-case cost for DCIS was C$ 14,505. The per-case cost averaged for all subtypes was C$ 39,263 and C$ 76,446, for stage I and II, respectively, rising to C$ 97,668 and C$ 370,398 for stages III and IV (Table 1, Figure 3). Costs varied widely by subtype, with a difference in cost up to 2.7-fold for the same stage of disease depending on subtype. TN and HR+ stage I were the least expensive to treat at C$ 25,247 and C$ 28,201. However, the cost of treatment for TN stage II rose dramatically and became the most expensive subtype to treat at this stage (C$ 101,811). HR+ and TN were the costliest for stage III (C$ 117,269; C$ 110,798), while HR+HER2+ and HER2+ were the most expensive for stage IV, at approximately C$ 515,000 per case. 

**Figure 4:**
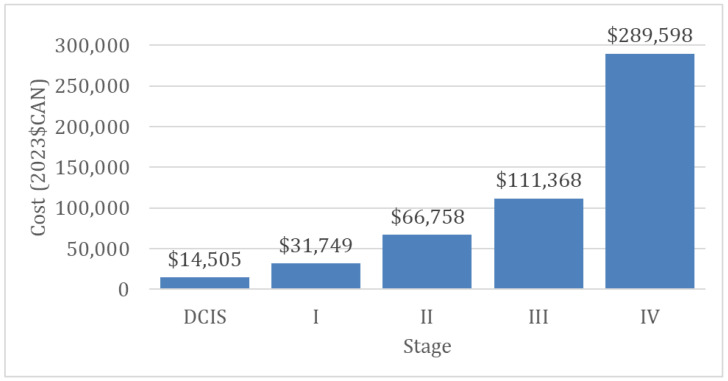
Cost of treatment in C$ by stage of breast cancer weighted by mean subtype proportion in Canadian women, excluding Quebec, ages 15–99, 2010–2017 diagnosis period.

From 2010 to 2017 in Canada, excluding Quebec, HR+ accounted for 74% of BC cases in the population, and HR+/HER+, HER2+ and TN made up 10.4%, 4.9% and 10.7%, respectively. Per-case treatment costs weighted by the proportion of subtype by stage were C$ 14,505, C$ 31,749, C$ 66,758, C$ 111,368 and C$ 289,598 for DCIS and stage I, II, III and IV (Figure 4).

Later-stage cancers were uniformly more expensive to treat than earlier-stage cancers. Stage IV cancers were between 7.7 (TN) and 10.9 (HER2+) times more expensive to treat than stage I cancers of the same subtype (Table 2). This discrepancy persisted between stage IV and stage II cancers, as metastatic cancers were 1.9–7.7 times more expensive to treat than stage II. Stage III cancers were also 1.5–4.2 times more expensive to treat than stage I cancers.

Costs broken down by diagnostic procedure or treatment type are shown in Table 3. Radiation and surgery costs varied little with subtype, while systemic therapy costs differed widely with subtype. Systemic therapy costs were the largest driver behind treatment expenses, constituting up to 90% of treatment costs. Variations in stage IV BC systemic therapy costs were primarily driven by novel therapeutics, e.g., immunotherapy and CDK4/6 inhibitors and differences in survival. Survival dictates the duration of treatment, and so accounts for the lower stage IV costs for TN BC, which had a survival of only 22 months compared to 66 months with HER2+ BC. 

## 4. Discussion

This ABC model allows for a real-time assessment of the per-case treatment of BC, customised to reflect subtype-specific treatment practices and survival patterns. The cost of treating BC rises exponentially with stage, with an up to 11-fold magnitude in cost savings with early diagnosis. The magnitude of cost difference increases to 36 times when DCIS is compared with stage IV. Costs are primarily driven by systemic therapy and survival time in stage IV disease, with fairly constant costs for diagnosis, surgical treatment and radiation therapy. HR+ BC accounts for approximately 74% of cases and will therefore preferentially influence treatment expenditures. The 26,175 BC cases diagnosed in Canada in 2017 would have cost C$ 1.8 billion to treat with appropriate stage and subtypes costs applied to these cases, not including the cost of treating recurrent disease.

Our ABC costs are significantly higher than costs determined in previous studies. Two-year costs from 2005 to 2009 in Ontario were (2012C$) C$ 29,938, C$ 46,893, C$ 65,369 and C$ 66,627 for stages I to IV, respectively [53]. Two recent studies considered the cost of treating ER+ and TN BC in Ontario based on cases from 2012 to 2016 [6,55]. The average annual per-patient treatment costs from these studies were (2017C$) C$ 22,662 (HR+) and C$ 35,064 (TN) for stage I–III and C$ 77,112 (HR+) and C$ 140,160 (TN) for stage IV; however, only 17% of patients with stage IV BC received CDK 4/6 inhibitors. The 10-year Medicare cost (2007–2016) of treating stage I BC was USD 136,428 and that for stage IV was USD 376,563, with cost being exponentially related to stage [56]. A 2021 micro-costing Netherlands model for lifetime hospital costs for advanced BC found an average cost of € 52,709 (C$ 77,482) ranging from € 29,803 (C$ 43,810) in TN to € 92,272 (C$ 135,639) in HR+/HER2+ BC [57]. A 2022 US time-based ABC did not present integrated case costs by treatment possibility, stage and subtype, but derived similar costs [58]. Mastectomy cost with implant was USD 12,129 (2022 USD) compared to our cost of C$ 9559 (2023C$), and USD 6481 (2022 USD) for 25 fractions of radiation compared to our cost of C$ 5337 (2023C$) for 15 fractions. However, the cost for stage III TN was less (2022 USD 27,864), primarily because immunotherapy (C$ 63,360) was not included in the US model. Although our per-case cost for radiation therapy was in agreement with the US timed-based study, it was lower (C$ 3682 vs. C$ 16,442, stage I) than that noted in a 2020 study from Ontario which used a costing algorithm to determine person-level costs [30]. This difference may partially be due to the recent shift to hypofractionated treatment regimes. 

ABC allows for broader inclusion of costs than is possible with a top-down approach. Our ability to include pathology, ongoing radiology and lab costs throughout treatment; facility and human resources costs through QBP funding; and drug costs which would not have been captured in public pay databases creates a more complete picture of the true cost of treating BC. Importantly, compared with top-down studies, there is no temporal lag in cost data, with all treatment costs representing 2023 costs and the most up-to-date practice patterns. Our ABC model accounts for rapidly evolving and costly new therapies such as immunotherapy and antibody–drug conjugates, which increase survival and therefore treatment duration. Additionally, the ABC approach is not curtailed by a specific time interval, so unlike other studies which reflect a one- or two-year timeframe for costs, we are able to account for costs for the total duration of treatment per case, up to seven years in stage III HR+ BC.

We have shown that the cost of BC treatment is many orders of magnitude less when BC is diagnosed at an earlier stage, and that costs increase exponentially with stage. The magnitude of difference in cost for treatment of late-stage compared to early-stage BC has been previously noted to be 3–4 times [55,56]; however, our study shows a cost differential of up to 36 times in magnitude when DCIS is compared with stage IV. These results suggest that the increased diagnosis of earlier-stage BC through breast cancer screening programs could result in significant cost savings to our health systems. A previous investigation of the cost-effectiveness of screening using the Oncosim model employed BC treatment costs from 2012, which limits applicability to current treatment practices [59]. Given the stage shift noted when women 40–49 are screened, our ABC costs should be used to re-examine screening cost-effectiveness [60]. 

There are limitations to our model. We did not model the lifetime cost per BC case. Thus, costs for the ongoing morbidity due to BC treatment were not included, and neither were the costs for retreatment of recurrent BC. A patient with stage III BC which recurs as metastatic disease could have a total treatment cost including that of stage III in addition to the cost of stage IV. Not every treatment assumption could be backed by RCT or published evidence. In these cases, expert opinion and institutional practice patterns were used, although we acknowledge that variability in clinician treatment preferences will always exist. We likely underestimated treatment costs as we did not include depreciation of equipment or all potential complications of BC and their associated costs, such as pathological fractures and thromboembolic disease. We also only included two years of hospitalisation, emergency and home care costs. Societal costs, such as lost productivity and time off work, are thought to represent up to 50% of medical treatment costs and were not included [61]. Finally, the drug and system costing is specific to Ontario, Canada. The inclusion of drugs within QBP funding and the funding of drugs through Cancer Care Ontario means that costs may be higher if treatment is delivered where similarly advantaged drug pricing is not available. However, the use of evidence-based guidelines and peer-reviewed literature to inform activity maps ensures that costs are generalisable beyond Ontario, and our derived costs are similar to those from ABC models from other jurisdictions.

This study finds that the cost of BC treatment far exceeds the costs found in previous studies. Published models do not accurately reflect the true costs of treating BC. One case of metastatic BC can cost up to C$ 516,415 (HR+/HER2+) for treatment, driven primarily by the cost of systemic therapy, compared to C$ 25,247 (TN) for stage I, a 20-fold increase in expense. The ABC model accurately reflects current treatment practices and can be simply updated on an ongoing basis so that future innovations in BC therapy can be readily incorporated. The costs from this ABC model should be used for health economics planning, and to determine the cost-effectiveness of screening given the inherent increase in the diagnosis of early-stage BC realised with screening.

## Figures and Tables

**Table 1:** Cost per treatment of case of breast cancer by subtype and stage. All costs in 2023C$.

Subtype	Stage				
	DCIS	I	II	III	IV
HR+	14,505	28,201	60,289	117,269	256,693
HR+/HER2+		C$ 56,401	76,547	86,653	516,415
HER2+		47,201	67,136	75,954	514,992
TN		25,247	101,811	C110,798	193,490
Mean	14,505	39,263	76,446	97,668	370,398

Abbreviations: DCIS = ductal carcinoma in situ, HR = hormone receptor, HER2 = her2 neu, TN = triple negative.

**Table 2:** Stage-to-stage ratio of treatment costs by subtype.

Subtype			Stage Comparison				
	DCIS vs. I	DCIS vs. II	DCIS vs. IV	III vs. I	III vs. II	IV vs. I	IV vs. II
HR+	0.5	0.2	0.1	4.2	1.9	9.1	4.3
HR+/HER2+	0.3	0.2	0.0	1.5	1.1	9.2	6.7
HER2+	0.3	0.2	0.0	1.6	1.1	10.9	7.7
TN	0.6	0.1	0.1	4.4	1.1	7.7	1.9
Mean	0.4	0.2	0.0	3.0	1.4	9.2	5.0

**Table 3:** Cost of treatment modalities for breast cancer case by stage and subtype. All costs in 2023C$.

Subtype	Treatment Modality	Stage				
		DCIS	I	II	III	IV
HR+	Diagnostic Imaging	C$ 0	C$ 169	C$ 169	C$ 424	C$ 6614
	Pathology	C$ 1813	C$ 4113	C$ 5755	C$ 2305	C$ 2116
	Surgery	C$ 6798	C$ 7808	C$ 7938	C$ 9436	C$ 1438
	Radiation Therapy	C$ 3682	C$ 3682	C$ 5337	C$ 4986	C$ 1711
	Systemic Therapy	C$ 2212	C$ 4307	C$ 27,665	C$ 80,162	C$ 209,228
	ER/Inpatient/Home care	C$ 0	C$ 8122	C$ 13,425	C$ 19,956	C$ 35,587
HR+/HER2+	Diagnostic Imaging		C$ 169	C$ 169	C$ 424	C$ 7788
	Pathology		C$ 1190	C$ 1190	C$ 2252	C$ 2116
	Surgery		C$ 7808	C$ 7938	C$ 9532	C$ 1438
	Radiation Therapy		C$ 3687	C$ 5401	C$ 4986	C$ 1856
	Systemic Therapy		C$ 35,425	C$ 48,423	C$ 49,503	C$ 467,631
	ER/Inpatient/Home care		C$ 8122	C$ 13,425	C$ 19,956	C$ 35,587
HER2+	Diagnostic Imaging		C$ 169	C$ 169	C$ 255	C$ 7788
	Pathology		C$ 1253	C$ 1253	C$ 2314	C$ 2116
	Surgery		C$ 7808	C$ 7938	C$ 9532	C$ 1438
	Radiation Therapy		C$ 3684	C$ 5401	C$ 4986	C$ 1914
	Systemic Therapy		C$ 26,166	C$ 38,949	C$ 38,910	C$ 466,150
	ER/Inpatient/Home care		C$ 8122	C$ 13,425	C$ 19,956	C$ 35,587
TN	Diagnostic Imaging		C$ 169	C$ 169	C$ 424	C$ 3202
	Pathology		C$ 1561	C$ 1561	C$ 2622	C$ 2572
	Surgery		C$ 7808	C$ 7938	C$ 9532	C$ 1438
	Radiation Therapy		C$ 3687	C$ 5401	C$ 4986	C$ 1923
	Systemic Therapy		C$ 3901	C$ 73,317	C$ 73,278	C$ 148,769
	ER/Inpatient/Home care		C$ 8122	C$ 13,425	C$ 19,956	C$ 35,587

## Data Availability

Data are available in the Supplementary Materials.

## Supplementary Materials

The following supporting information can be downloaded at: https://www.mdpi.com/article/10.3390/curroncol30090571/s1, Table S1: Activity-based costing model for breast cancer by stage and molecular subtype.
